# Assessing lung aeration using ultrasound after birth in near-term lambs at risk of respiratory distress

**DOI:** 10.3389/fped.2023.1148443

**Published:** 2023-05-22

**Authors:** E. J. Pryor, I. M. Davies, K. J. Crossley, A. M. Thiel, E. V. McGillick, K Rodgers, I Nitsos, M. J. Kitchen, D. A. Blank, S. B. Hooper

**Affiliations:** ^1^ The Ritchie Centre, Hudson Institute of Medical Research, Melbourne, VIC, Australia; ^2^ Department of Obstetrics and Gynaecology, Monash University, Melbourne, VIC, Australia; ^3^ School of Physics and Astronomy, Monash University, Melbourne, VIC, Australia; ^4^ Monash Newborn, Monash Children’s Hospital, Clayton, VIC, Australia

**Keywords:** lung ultrasound, lung ultrasound (LUS), neonate, respiratory distress at birth, respiratory distress - transient tachypnoea of newborn or 'wet lung syndrome', support

## Abstract

**Background:**

Optimizing respiratory support after birth requires real-time feedback on lung aeration. We hypothesized that lung ultrasound (LUS) can accurately monitor the extent and progression of lung aeration after birth and is closely associated with oxygenation.

**Methods:**

Near-term (140 days gestation, term ∼147 days), spontaneously breathing lambs with normal (controls; *n* = 10) or elevated lung liquid levels (EL; *n*= 9) were delivered by Caesarean section and monitored for four hours after birth. LUS (Phillips CX50, L3–12 transducer) images and arterial blood gases were taken every 5–20 min. LUS images were analyzed both qualitatively (grading) and quantitatively (using the coefficient of variation of pixel intensity (CoV) to estimate the degree of lung aeration), which was correlated with the oxygen exchange capacity of the lungs (Alveolar-arterial difference in oxygen; AaDO_2_).

**Results:**

Lung aeration, measured using LUS, and the AaDO_2_ improved over the first 4 h after birth. The increase in lung aeration measured using CoV of pixel intensity, but not LUS grade, was significantly reduced in EL lambs compared to controls (*p* = 0.02). The gradual decrease in AaDO_2_ after birth was significantly correlated with increased lung aeration in both control (grade, r^2 ^= 0.60, *p* < 0.0001; CoV, r^2 ^= 0.54, *p* < 0.0001) and EL lambs (grade, r^2 ^= 0.51, *p* < 0.0001; CoV, r^2 ^= 0.44, *p* < 0.0001).

**Conclusions:**

LUS can monitor lung aeration and liquid clearance after birth in spontaneously breathing near-term lambs. Image analysis techniques (CoV) may be able detect small to moderate differences in lung aeration in conditions with lung liquid retention which are not readily identified using qualitative LUS grading.

## 1. Introduction

During fetal development the lungs are liquid-filled, which is essential for normal lung growth and development (1). At birth, this liquid must be cleared quickly in order to establish pulmonary gas exchange and facilitate a healthy transition from fetal to newborn life. Over 5% of all newborns require assistance to aerate their lungs, but the optimal type of assistance is unknown and likely varies between individuals (2). To optimise respiratory support in individual infants immediately after birth, real-time feedback is required to monitor the extent and progression of lung aeration.

**Figure 1:**
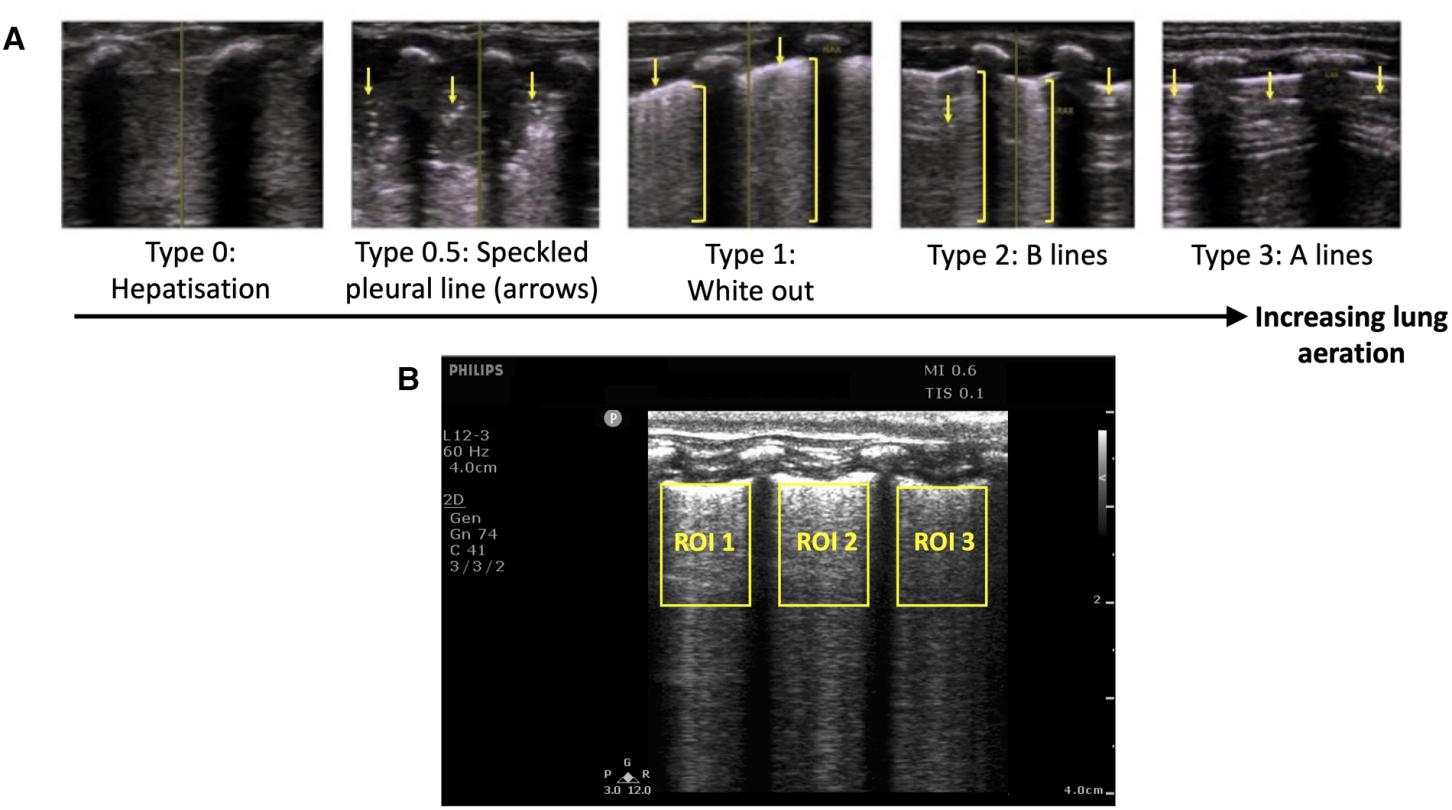
(**A**) Grading system used to describe the evolution of lung ultrasound images during the transition to air-breathing adapted from Raimondi et al. (12) as previously used by Blank et al. (13) Type 0: “hepatisation,” the ultrasound beam does not encounter air, as it passes through liquid and soft tissue. The pleural line is either extremely thin or hypoechoic. Type 0.5: “speckled” pleural line, patchy hyperechoic appearance with poor definition and is not horizontal in orientation. This image is transiently visible in neonates after the initiation of breathing, but before the establishment of the pleural line. Type 1: “white-out lung”, associated with respiratory distress syndrome. Type 2: Vertical “B lines” arising from the pleural line (brackets), with areas of horizontal A-lines (arrows). Type 3: horizontal “A-lines”, encountered when the US beam bounces between an aerated lung and the US transducer. (**B**) Example lung ultrasound image with three regions of interest (ROI; area below the pleural line, not including acoustic shadows) selected. The coefficient of variation of pixel intensity (LUS CoV) was calculated from these ROIs, and used to estimate the proportion of air in the lungs as previously described (14).

Lung ultrasound (LUS) is a promising imaging modality which can accurately diagnose respiratory conditions in neonates at the bedside (3–6), predict infants at risk of deteriorating (7–10) and may be a valuable tool in guiding treatment decisions (11). Furthermore, LUS may be used to assess the degree of lung aeration in newborn infants and provide feedback information on lung function. The type and quantity of different LUS imaging artifacts varies as the lung progressively aerates and penetration of the ultrasound beam through lung tissue is gradually reduced by the increasing presence of air. LUS images have previously been scored based on the presence or absence of different image artifacts, which provide a rough indication of the degree of lung aeration (Figure 1). These imaging artifacts have been well characterised and correlate well with the amount of air in the lungs of newborn lambs (14). Indeed, LUS can describe changes during the transition to air-breathing at birth in healthy term infants (12, 13, 15, 16), term infants with respiratory distress (12), and preterm infants (12, 17). While LUS is a promising candidate for assessing lung aeration, studies examining the utility of LUS in infants during the transition to air-breathing have been limited to 1–4 ultrasound scans in the first few hours after birth. However, this is insufficient to accurately relate changing LUS image characteristics with changing lung function during lung aeration, particularly as LUS images can be quite variable during this period (13, 15, 17). One of our aims was to determine how closely the changing LUS images that result from lung aeration after birth reflect improving (or worsening), physiological measures of lung function. We hypothesised that the changes in LUS images are closely associated with a measure of the lung's oxygen exchange capacity (Alveolar-arterial difference in oxygen; AaDO_2_) in spontaneously breathing control lambs and in lambs at risk of developing respiratory distress shortly after birth due to the presence of elevated lung liquid levels.

Previous studies investigating the evolution of LUS images in human infants have shown that in some infants, the lung may appear well aerated lung in the first 10 min after birth, but is less well aerated on a subsequent examination performed 1–3 h after birth (13, 15, 17). This phenomenon, termed “backsliding”, is common and occurs in approximately half of both preterm (17) and term infants (15). Importantly, preterm infants with “backsliding” tended to have higher rates of intubation and surfactant therapy following delivery (17). The phenomenon of backsliding has not been well described and the underlying pathology is largely unknown, but it may indicate which infants require intervention and might prevent these infants from deteriorating. A second aim of this study was to investigate factors associated with “backsliding” and its relationship with lung oxygen exchange capacity (AaDO_2_) in spontaneously breathing near-term neonatal lambs. We hypothesised that backsliding may occur when breathing patterns change (such as vigorous into quiet breathing) or when the ventilatory strategy changes (such as invasive to non-invasive ventilation) and is associated with worsening AaDO_2_.

## 2. Methods

### 2.1. Ethical approval

All experimental procedures were approved by the Monash Medical Centre Animal Ethics Committee and Monash University. All experiments were conducted in accordance with the National Health and Medical Research Council (NHMRC) Australian code of practice for the care and use of animals for scientific purposes.

### 2.2. Delivery and initial respiratory management

Previously instrumented lambs (18) were delivered via Caesarean section under spinal anaesthesia at 140 days gestation (term ∼147 days, equivalent to ∼37 weeks human lung development). Ewes were sedated (propofol 1%; 20–40 mg bolus IV) prior to injection of lignocaine 2% (0.1 ml/kg) to induce spinal anaesthesia via the lumbosacral space. Following induction of the spinal block, mild sedation of the ewe continued using midazolam (1 mg/kg/hr IV) before the fetus was exteriorised, leaving the umbilical cord intact. Before the onset of breathing, as much lung liquid as possible was drained via the indwelling tracheal catheter, to mimic lung liquid loss during vaginal delivery and standardise lung liquid levels between lambs. Lambs allocated to the elevated liquid (EL; *N* = 9) group had 20 ml/kg of Hartmann's solution added back into the lungs via the indwelling tracheal catheter before the onset of breathing (to mimic the volume of lung liquid expected after caesarean section without labour), while control lambs only had lung liquid drained (*N* = 10, to mimic volume of lung liquid expected after vaginal delivery).

Lambs were then stimulated to breathe and were provided with initial respiratory support while still attached to the umbilical cord. Flumazenil (0.01 mg/kg IV) and naloxone (0.01 mg/kg IV) was administered to each lamb to block any suppressive effects of maternal midazolam and fentanyl on breathing that may have crossed the placenta. The lamb was stimulated to breathe, but if regular and stable breathing did not begin or if the lamb displayed poor respiratory efforts, caffeine (20 mg/kg IV, up to two doses) was given and intermittent positive pressure ventilation was applied non-invasively, while the lamb was still attached to the cord. If the lamb experienced ongoing apnoeas despite receiving continuous positive airway pressure, it was intubated and mechanically ventilated until it displayed regular spontaneous breathing. Once regular breathing patterns were established, the umbilical cord was clamped and the lamb was transferred to a custom-made lamb sling under a radiant heater.

### 2.3. Ongoing management and physiological recordings

Lambs were monitored for four hours after delivery. Oxygen/air mixture was provided using custom made nasal prongs at a rate of up to 15 L/min if required, which was used to provide some end-expiratory pressure in the absence of an effective seal around the prongs. The gas flow rate and fraction of inspired oxygen (FiO_2_) were adjusted to maintain oxygen saturations (from arterial blood gas readings) above 90%. Body temperature was measured using a rectal thermometer and maintained at ∼39°C. A maintenance glucose infusion was initiated shortly after delivery (Glucose 5%, IV, 6 ml/kg/hr) and lambs received regular enteral feeds. Arterial blood gas samples were taken regularly after cord clamping from a left carotid arterial catheter (every 5 min until 30 min after birth, then every 10 min until 60 min after birth, and every 20 min thereafter). Electronic recordings of intrapleural and upper tracheal pressure were acquired continuously throughout the experiment using LabChart software (Powerlab ADI, Sydney, Australia), from catheters inserted into the intrapleural space and the upper trachea during fetal instrumentation surgery performed at 137 days gestation, as previously described (18).

### 2.4. Lung ultrasound image acquisition

LUS images were acquired as 3-s DICOM clips with a Phillips CX-50 ultrasound machine and an L3–12 linear transducer, with a depth of 4 cm, the focus set at the pleural line, a frame rate of 15 images per second, a gain of 74 and harmonics turned off. This transducer has a frequency range of 3–12 MHz. Images were acquired before the onset of breathing, every 2.5 min during the first 10 min after the onset of breathing, then every 5 min until 30 min after birth, every 10 min until 60 min after birth, and every 20 min thereafter, until the end of the experiment (4 h after birth). Images were also acquired within 5 min if the gas flow rate was changed. Where possible, the lamb was imaged in two locations (usually the centre of right and left lungs). A 3 × 3 cm area of the lamb's chest was shaved on both sides after the lamb was moved to the custom sling to ensure the imaging location was kept consistent between scans.

### 2.5. Changes to oxygen/air flow rate at the end of the experiment

At the end of the experiment, the oxygen/air gas flow was ceased for two minutes and then increased to 15 L/min for ∼2 min to determine the effect of changing ventilation support on lung function and LUS images, which were acquired ∼2 min before and after the flow rate was changed.

### 2.6. Lung ultrasound image analysis

Lung ultrasound images were analysed using the following techniques:

1. LUS grade: All LUS images were graded by one researcher (EP) using a previously described grading scale (12, 15) (Figure 1). A random sample of 92 images was graded by a second blinded researcher (DB), and the inter-rater reliability was calculated and reported as a percentage.
2. LUS coefficient of variation (CoV) of pixel intensity: The estimated proportion of air (EPA) in the lungs was calculated using a recently described technique (14). This technique involves manually defining rectangular regions of interest in each ultrasound recording, defined as any area of lung below the pleural line and between acoustic shadows formed by the ribs (Figure 1). Frames coinciding with large amounts of movement were excluded from this analysis. The mean CoV in these regions was calculated by dividing the standard deviation of pixel intensity by the mean pixel intensity, and a simple linear regression model was used to determine the EPA from the CoV as previously described (14). The EPA ranges from 0 (lung is completely liquid filled) to 1 (lung is completely air-filled).

Backsliding was defined as either: (i) a reduction in the LUS grade, or (ii) a reduction in the EPA by >0.2 in either lung, from one time point to the next time point. Severe backsliding was defined as (i) a reduction in LUS grade to grade 1 or worse, or (ii) a reduction in the EPA in either lung by >0.2, to an average EPA (in both lungs) of <0.6.

### 2.7. Physiological parameters

5 second averages of the respiratory rate, upper tracheal pressure and intrapleural pressure were analysed at the time (±1 min) ultrasound images were acquired, taking care to avoid recordings that included artefacts associated with lamb movement or vocalisation.

If lambs were spontaneously breathing and provided with air/oxygen gas flow rates of >4 L/min during the 4 h experiment, LUS images and 30-s averages of physiological parameters (respiratory rate, intrapleural and upper tracheal pressure), and the variability in respiratory rate (the coefficient of variation of breath length during each 30-s sequence), were compared both before and after the flow rate was changed. This was used to assess whether changing the flow rate affected respiratory physiology or the appearance of the LUS images.

The alveolar-arterial difference in oxygenation (AaDO_2_, mmHg) was calculated as previously described from arterial blood gas results using the formula: FiO_2_ × (760 – saturated water vapor pressure) − PaCO_2_/0.8 − PaO_2_, where units are all in mmHg, 760 mmHg was used as the mean atmospheric pressure within our laboratory, saturated water vapor pressure was calculated for the lamb’s body temperature (measured from a rectal thermometer, 52 mmHg at 39°C), and 0.8 is an assumed respiratory quotient (19).

### 2.8. Statistical analysis

Statistical analysis was performed in GraphPad Prism 9. Ordinal variables (LUS grade) were presented as median (interquartile range). Numerical variables (EPA, intrapleural pressure, upper tracheal pressure, respiratory rate, AaDO_2_, number of backsliding occurrences) were tested for normality with a Shapiro-Wilk test and were presented as mean ± standard error of the mean (SEM) if normally distributed, or median (interquartile range) if not normally distributed.

LUS grades between control and EL lambs were compared at each time point using Mann-Whitney *U* tests (adjusted for multiple comparisons using Bonferroni correction), and the EPA was compared using a mixed-effects model. Multivariate linear regression was used to describe the relationship between the AaDO_2_ and LUS grade and EPA within each lamb (20). The respiratory rate, variability (coefficient of variation) of breath length, and respiratory effort (change in intrapleural pressure from end-inspiration to end-expiration) were calculated from 5-breath blocks acquired immediately before and for 30 breaths after the gas flow rate was increased to 15 L/min at the end of the experiment. Values after the flow rate was increased were compared to the value before the flow rate was increased using a mixed effects analysis with Dunnet's test for multiple comparisons.

The intrapleural pressure, upper tracheal pressure and EPA was compared before and after the type of respiratory support or gas flow rate was changed using a paired *t*-test (if data were normally distributed) or Wilcoxon test (if not normally distributed); LUS grade was compared using a Wilcoxon test.

The number of backsliding occurrences were compared between control and EL lambs using an independent samples *t*-test when normally distributed or Mann-Whitney *U* test if not normally distributed. Physiological parameters (intrapleural pressure, upper tracheal pressure, respiratory rate, AaDO_2_) were compared before and after backsliding was observed using a paired *t*-test when normally distributed or Wilcoxon test if not normally distributed.

## 3. Results

Results are presented for a total of 19 lambs (control, *N* = 10; EL, *N* = 9). The inter-rater reliability for the LUS grade in a random sample of 92 images was 82%.

**Figure 2:**
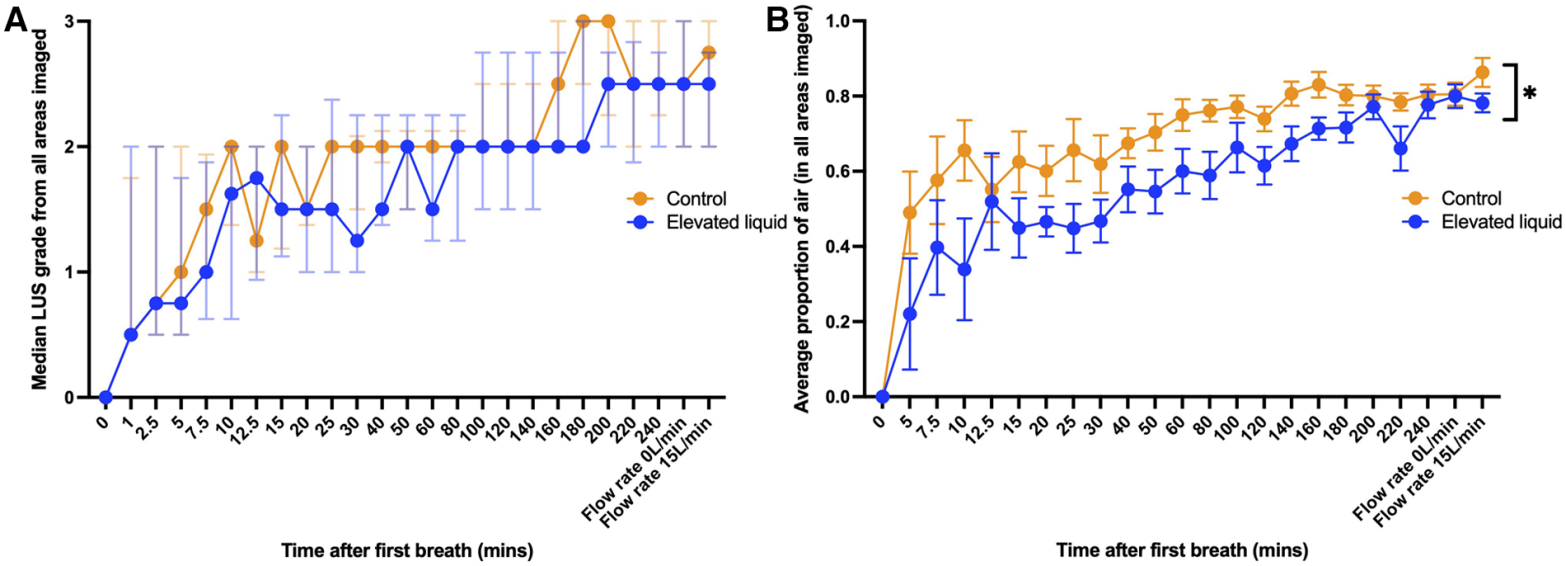
(**A**) Median (IQR) LUS grade (in all regions imaged) over time between control lambs (*N* = 10) and lambs with elevated lung liquid levels at birth (*N* = 9; Mann-Whitney *U* tests with Bonferroni correction for multiple comparisons, *p* > 0.05 at every time point). (**B**) Mean (±SEM) estimated proportion of air in the lungs (in all regions imaged) over time between control lambs and lambs with elevated lung liquid levels at birth. While lung aeration improved in both groups over time, lambs with elevated lung liquid levels at birth had a consistently lower estimated proportion of air in their lungs throughout the experiment (repeated measures mixed effects analysis, main effect of time *p* < 0.0001, main effect of group *p* = 0.02).

Both the LUS grade and the EPA increased progressively in both control and EL lambs over time after the onset of air-breathing (Figure 2). Control lambs had a consistently higher EPA than EL lambs during lung aeration (Figure 2; *p* = 0.02). Similarly, while there was no significant difference in the median LUS grade between control and EL lambs at any time point, the LUS grade tended to be higher in control than in EL lambs, indicating a higher degree of lung aeration.

**Figure 3:**
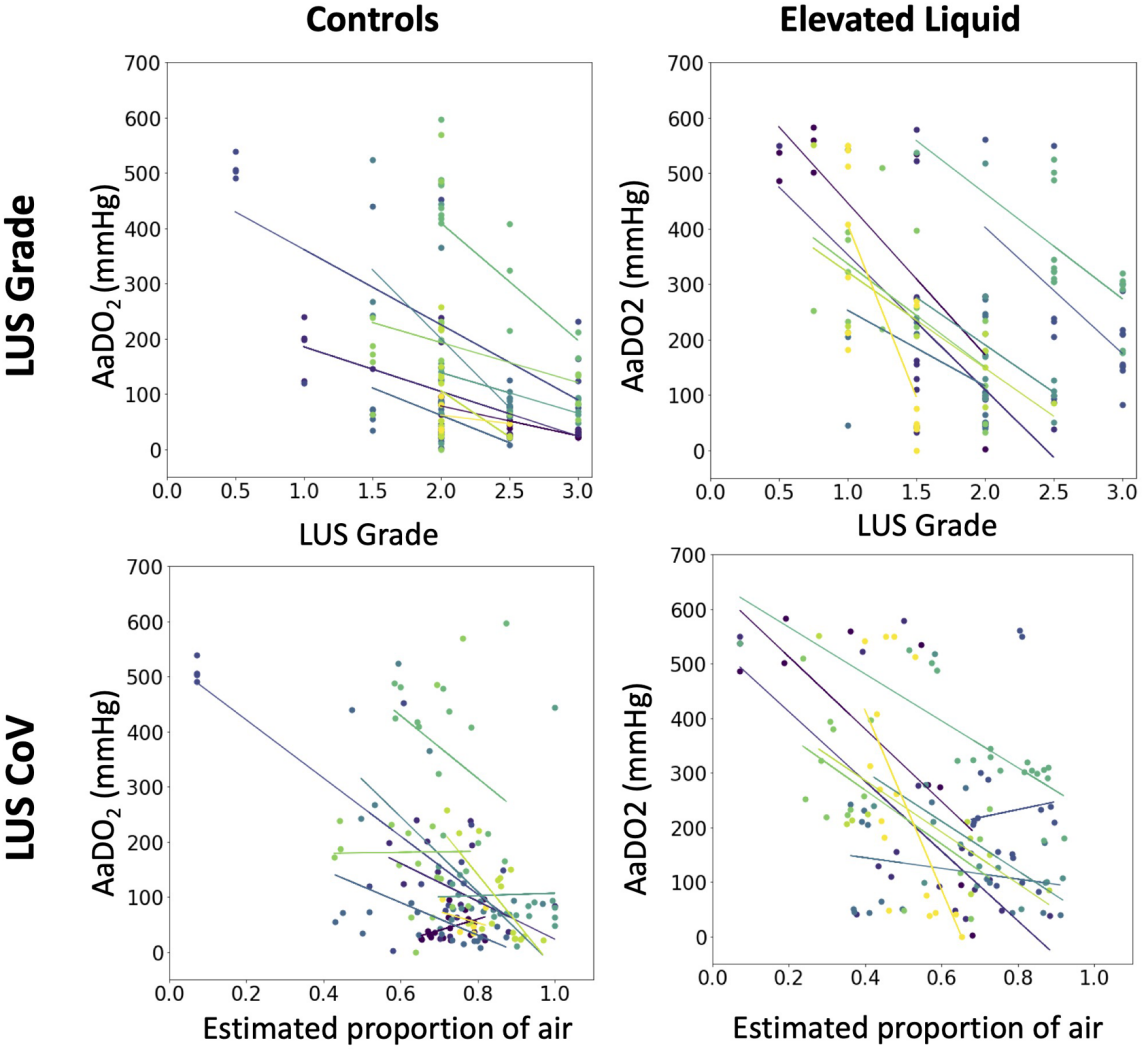
The relationship in each lamb between the alveolar-arterial oxygen gradient (mmHg) and the median lung ultrasound grade (multivariate linear regression, controls, *N* = 10, r^2 ^= 0.60, *p* < 0.0001; EL, *N* = 9, r^2 ^= 0.51, *p* < 0.0001), and the estimated proportion of air in the lungs (multivariate linear regression, controls, *N* = 10, r^2 ^= 0.54, *p* < 0.0001; EL, *N* = 9, r^2 ^= 0.44, *p* < 0.0001).

In both control and EL lambs, the AaDO_2_ significantly decreased during lung aeration and the increase in both LUS grade and the estimated proportion of air were significantly correlated with the decrease in AaDO_2_ (Figure 3) after the onset of air-breathing (controls, LUS grade, r^2 ^= 0.60, *p* < 0.0001; EL, LUS grade, r^2 ^= 0.51, *p* < 0.0001; controls, EPA, r^2 ^= 0.54, *p* < 0.0001; EL, EPA, r^2 ^= 0.44, *p* < 0.0001).

A total of eight lambs (3 controls, 5 EL) required intubation during resuscitation. A total of 17 lambs (9 controls, 8 EL) were provided with air/oxygen flow rates of >4 L/min during the experiment and the flow rate was decreased by >4 L/min 33 times in total across these 17 lambs. Reducing the air/oxygen flow rate resulted in a small reduction in the upper tracheal pressure (at peak inspiration, −1.31 ± 0.42 cmH_2_O, *p* < 0.01; at end of expiration, −1.12 ± 0.55, *p* = 0.05), however, neither reducing the air/oxygen flow rate nor extubation resulted in a significant change to the LUS image (Table 1).

**Table 1:** Changes in lung ultrasound and physiological parameters when the method of respiratory support was altered. The intrapleural pressure, upper tracheal pressure and estimated proportion of air was compared before and extubation or a decrease in gas flow rate a paired *t*-test (if data were normally distributed) or Wilcoxon test (if not normally distributed) and LUS grade was compared using a Wilcoxon test.

	Tracheal pressure (cmH_2_O)		Intrapleural pressure (cmH_2_O)		Lung ultrasound	
	At peak inspiration	At end of expiration	At peak inspiration	At end of expiration	LUS grade	Estimated proportion of air
Extubation (*N* = 9 occurrences)	−0.01 ± 1.72 (*p* = 0.99)	3.50 ± 2.63 (*p* = 0.23)	−0.61 ± 1.81 (*p* = 0.74)	−0.86 ± 3.68 (*p* = 0.82)	0 (−0.25–0.13) (*p* = 0.99)	−0.02 ± 0.04 (*p* = 0.72)
Decreasing flow rate by >4 L/min (*N* = 33 occurrences)	−1.31 ± 0.42 (*p* < 0.01)**	−1.12 ± 0.55 (*p* = 0.05)	−0.79 ± 0.49 (*p* = 0.12)	0.16 ± 1.28 (*p* = 0.90)	0 (0–0.25) (*p* = 0.55)	0.00 ± 0.03 (*p* = 0.91)

** *p* < 0.01.

**Figure 4:**
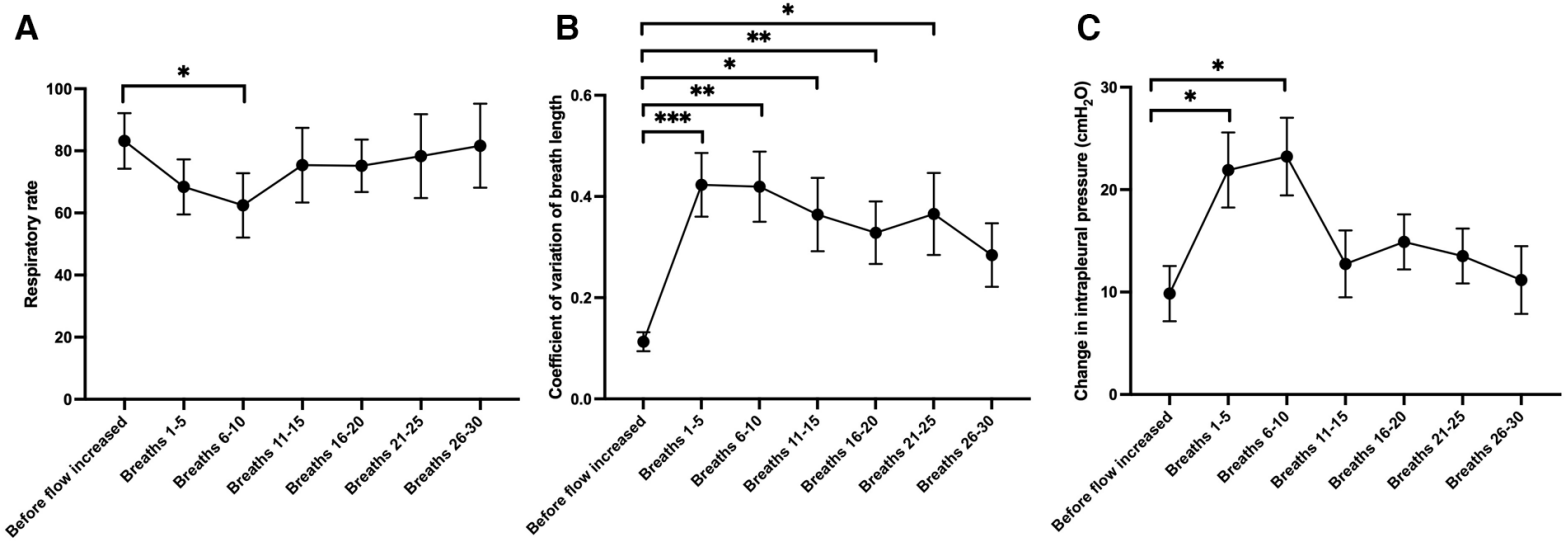
Changes in (**A**) respiratory rate, (**B**) the coefficient of variation of the length of each breath (a marker of the regularity of breathing) and (**C**) the change in intrapleural pressure from end-inspiration to end-expiration (a marker of respiratory effort), during 5-breath periods after the gas flow rate was increased from 0 L/min to 15 L/min at the end of the experiment (*N* = 17 lambs). Mixed effects model with Dunnett test for multiple comparisons; *p < 0.05, **p < 0.01, ***p < 0.001 (compared to the value acquired before flow rate was increased).

The air/oxygen flow rate was increased from 0 L/min to 15 L/min at the conclusion of the experiment in 17 lambs (8 controls, 9 EL). While the upper tracheal pressures at peak inspiration were observed to increase when the flow rate was increased (3.77 ± 0.92 cmH_2_O, *p* < 0.01), there was no significant change in the LUS grade (*p* = 0.79) or the EPA (increase of 0.03 ± 0.02, *p* = 0.23). The respiratory rate substantially decreased soon after the flow rate was increased (by 20.74 ± 6.70 breaths per minute, 6–10 breaths after the flow rate was changed; Figure 4A, *p* = 0.03) and three 3 lambs developed apnoeas. Similarly, respiratory effort increased, as evidenced by a larger intrapleural pressure differential between end-inspiration and end-expiration (increase of 13.4 ± 4.6 cmH_2_O, 6–10 breaths after the flow rate was changed, Figure 4C, *p* = 0.05). The respiratory rate was substantially more variable after the flow rate was increased (increase in coefficient of variation of breath length by 31% ± 7%, 6–10 breaths after the flow rate was changed, Figure 4B, *p* = 0.003). The breathing pattern in most lambs stabilised within 30 breaths (Figure 4) and, by this point, breathing parameters were not significantly different from the period before increasing the flow rate.

Backsliding was common in both control and EL lambs, regardless of how it was defined (Table 2). A reduction in LUS grade occurred in every lamb at least once during the experiment, and often occurred multiple times (average of 3.00 ± 0.49 occurrences in control lambs and 3.78 ± 0.80 occurrences in EL lambs, *p* = 0.41). Severe backsliding occurred in more than half of lambs and was occasionally observed to occur twice or more throughout the experiment. While severe backsliding (to a grade 1 image or worse) was observed to occur slightly more often in EL lambs, this was not statistically significant (average of 2.00 ± 0.42 occurrences in control lambs and 2.44 ± 0.65 occurrences in EL lambs, *p* = 0.052). Severe backsliding was associated with a small but significant decrease in the measured intrapleural pressure in EL lambs (at peak inspiration, 2.11 ± 0.96 cmH_2_O, *p* = 0.04; at end of expiration, −0.28 (−2.49 to 0.15) cmH_2_O, *p* = 0.03). Backsliding was not associated with a change in the AaDO_2_, upper tracheal pressure or respiratory rate (Table 3).

**Table 2:** Description of backsliding occurrences in control lambs and lambs with elevated lung liquid levels at birth. The average number of backsliding occurrences per experiment (mean ± SEM) was compared between control lambs and lambs with elevated airway liquid levels (EL) at birth using an independent samples *t*-test if normally distributed or Mann-Whitney *U* test if not normally distributed.

	Backsliding		Severe backsliding	
	LUS grade	Estimated proportion of air	LUS Grade	Estimated proportion of air
Backsliding observed, controls	10/10 (100%)	9/10 (90%)	7/10 (70%)	6/10 (60%)
Average number of occurrences, controls	3.00 ± 0.49	1.20 ± 0.33	2.00 ± 0.42	0.80 ± 0.29
Backsliding observed, elevated liquid	9/9 (100%)	7/9 (78%)	7/9 (78%)	6/9 (67%)
Average number of occurrences, EL	3.78 ± 0.80	2.44 ± 0.65	2.44 ± 0.65	2.00 ± 0.60
*p* value (number of occurrences, controls vs. EL)	0.41	0.57	0.05	0.14

**Table 3:** Change in physiological parameters observed when severe backsliding occurred (reduction in lung ultrasound grade to type 1 or worse). A paired t test (or Wilcoxon test) was performed, comparing the value of each parameter at the time point immediately backsliding occurred, compared to the time point when backsliding was observed. Data is presented as mean (SEM) of the differences between time points, or median (IQR) of the differences between time points if data were not normally distributed.

	Controls (*N* = 13 occurrences)	Elevated liquid (*N* = 24 occurrences)
AaDO_2_ (mmHg)	−46.58 ± 61.04 (*p* = 0.48)	−5.20 (−39.75 to 63.97), *p* = 0.76
Intrapleural pressure at peak inspiration (cmH_2_O)	−0.82 (−3.97 to 2.17), *p* = 0.47	−2.11 ± 0.96, *p* = 0.04*
Intrapleural pressure at end of expiration (cmH_2_O)	−0.55 (−4.39 to 1.38), *p* = 0.30	−0.28 (−2.49 to 0.15), *p* = 0.03*
Upper tracheal pressure at peak inspiration (cmH_2_O)	1.00 ± 0.89, *p* = 0.29	0.43 ± 0.51, *p* = 0.68
Upper tracheal pressure at end of expiration (cmH_2_O)	0.88 (−1.77 to 2.74), *p* = 0.49	−0.61 (−3.08 to 1.78), *p* = 0.38
Respiratory rate	11.46 ± 9.45, *p* = 0.18	7.64 ± 21.33, *p* = 0.19

* *p* < 0.05.

## 4. Discussion

In this study we have assessed the utility of LUS to monitor lung aeration during the immediate newborn period in spontaneously breathing neonatal lambs, with a low (control) or high (EL) risk of developing respiratory distress. We saw a clear improvement in the LUS images over the first four hours after air-breathing onset in both control and EL lambs. These increases were associated with increases in respiratory gas exchange across the lung (assessed using AaDO_2_). When LUS images were analysed using the CoV of pixel intensity, which estimates the proportion of air in the lungs, the trajectory of lung aeration and liquid clearance was clearly slower in lambs with elevated liquid levels at birth. ‘Backsliding’ was common, but the rate of backsliding was similar in both control and EL lambs.

**Figure 5:**
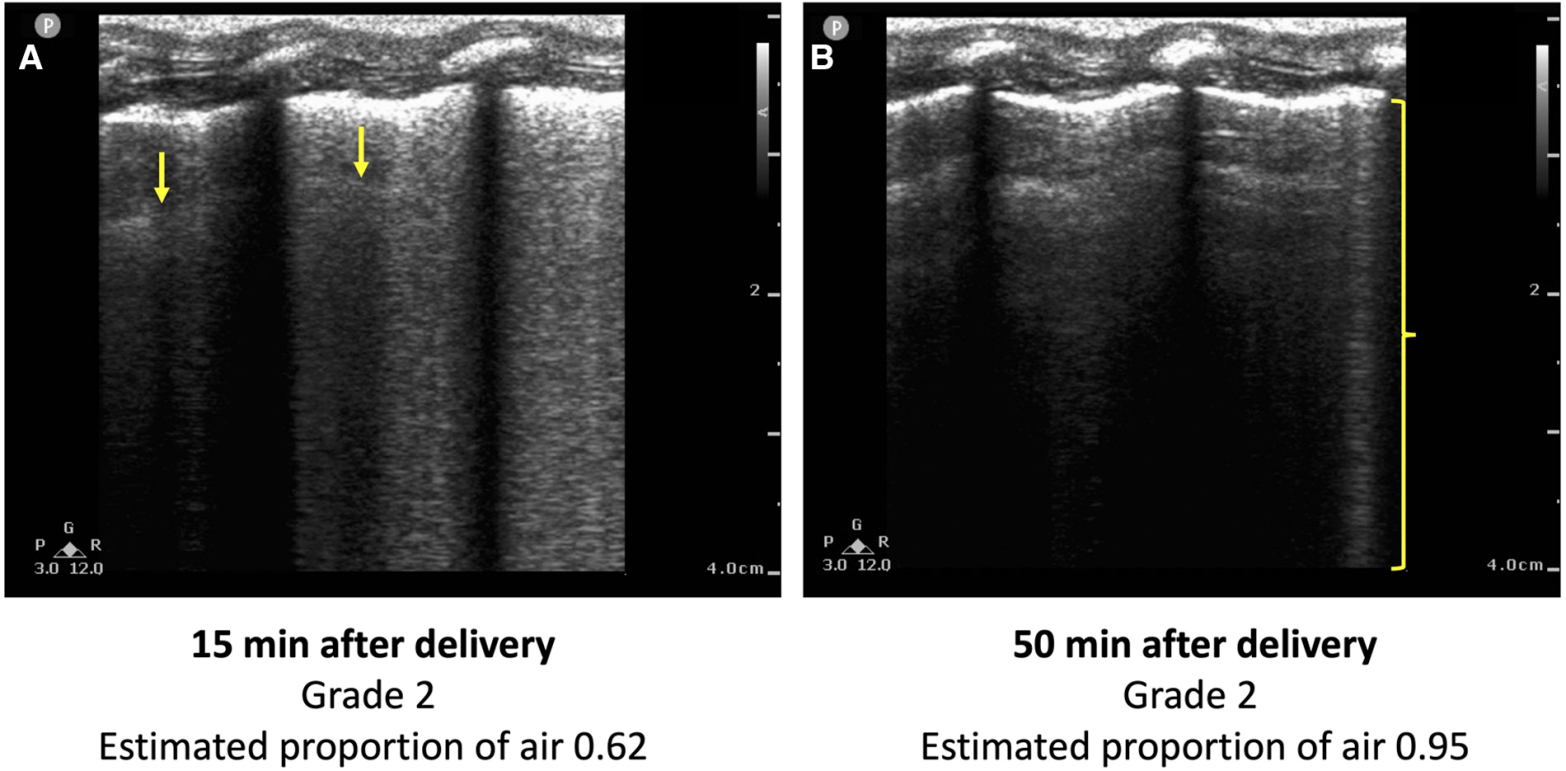
Two lung ultrasound images acquired from the same lamb (**A**) 15 min and (**B**) 50 min after the onset of breathing. The image is predominated by coalescence of B lines, which are associated with retained lung liquid and RDS. One B line is visible in (**B**) (bracket), but the image is predominated by A lines. Using conventional LUS grading systems, both images would be classified as a type 2, despite (**B**) indicating a higher degree of lung aeration than (**A**). The estimated proportion of air, calculated using a previously developed technique from the coefficient of variation of pixel intensity, is greater in (**B**) (0.95 vs. 0.62).

The EPA, but not the LUS grade, was significantly different between control and EL lambs, particularly during the first three hours after delivery. Current LUS grading scales (both LUS grade and LUS score) involve assigning the images a grade based on the presence of different artifacts, particularly A lines and B lines. This does not take into account the fact that the quantity and brightness of the B lines also reduces as the lung progressively aerates. For example, both LUS images in Figure 5 would be given a grade of 2, despite the image acquired at 50 min after air-breathing onset having considerably fewer B lines, indicating better lung aeration, than the image acquired at 15 min. In contrast, using the LUS image analysis technique (CoV) we developed to calculate the estimated proportion of air in the lungs (EPA) (14), we can clearly differentiate the difference in lung aeration between the two images shown in Figure 5. Thus, it appears that grading LUS images is not always capable of detecting small to moderate differences in lung aeration, whereas using the CoV to calculate the EPA can detect these moderate differences in lung aeration.

As expected, the alveolar-arterial difference in oxygen (AaDO_2_) improved as both the LUS grade and the EPA increased (Figure 3). Similar observations have been made in clinical studies in infants with respiratory pathology who are hours to days old; infants with worse LUS images tended to have a higher AaDO_2_ (8, 21). However, to our knowledge, the ontogeny of the changes in LUS images in relation to respiratory gas exchange has not been examined during lung aeration in the first minutes to hours after birth. The AaDO_2_ measures the difference in oxygen concentration between alveoli and arterial blood, with a lower AaDO_2_ (approaching 0) indicating that oxygen can diffuse easily across the epithelial membrane. In newborn lambs, a higher AaDO_2_ may occur because the presence of liquid in the airways reduces the capacity for oxygen to diffuse across the epithelial membrane. Observing a correlation between lung aeration (measured using LUS) and the AaDO_2_ further validates that both the LUS grade and LUS CoV can monitor lung aeration and the lungs increasing gas exchange potential over time.

**Figure 6:**
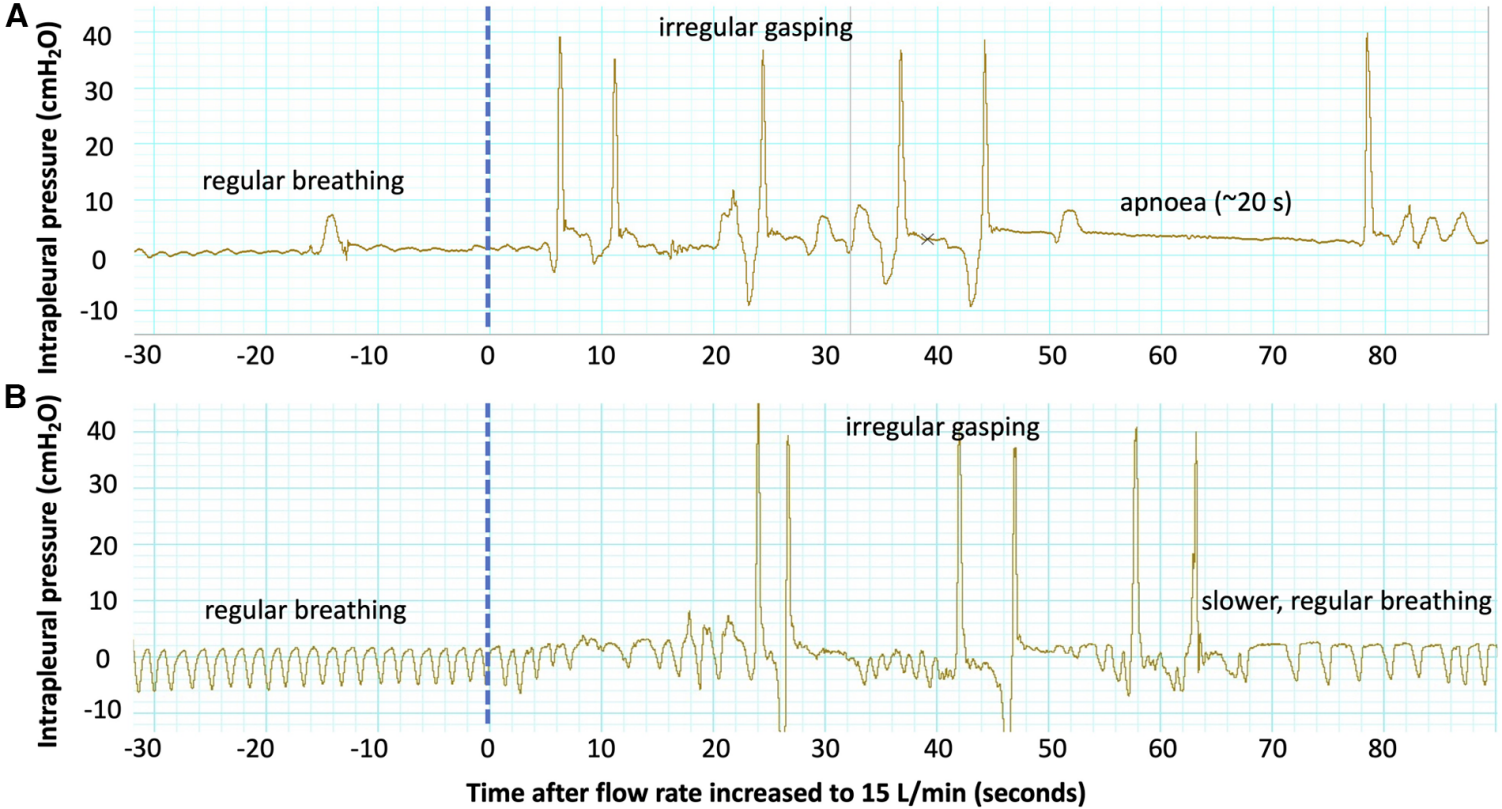
Examples of intrapleural pressure recordings from two lambs showing changes in the breathing pattern after the flow rate was increased to 15 L/min at the end of the experiment. Both lambs demonstrate regular, quiet breathing (regular rate and low amplitude) before the flow rate is increased (dotted blue line). Immediately after the flow is increased, both lambs experience irregular gasping with an increased respiratory effort (high amplitude). The lamb in (**A**) then experiences a ∼20 s apnoea, and the breathing pattern remains irregular until the end of the experiment. The lamb in (**B**) establishes regular breathing again 60 s after the flow rate is increased, though the respiratory rate is reduced.

The LUS image was not observed to improve when the air/oxygen flow rate was increased from 0 L/min to 15 L/min at the end of the experiment. Since increasing the flow rate increased the pressure in the distal airways, as evidenced by an increase in upper tracheal pressures, we expected lung aeration to improve. Note that a sample size of 17 lambs would be powered to detect a small change in the EPA of 0.075 after increasing the flow rate to 15 L/min, assuming a standard deviation in the EPA of 0.1 (from experimental data) and given a statistical power of 80%. Our inability to detect any improvement in lung aeration may be because the process of lung liquid clearance was likely complete in most lambs by four hours after delivery. Indeed, by four hours after air-breathing onset, all lambs had achieved a consistent image grade of 2 or 3 in both lungs and the estimated proportion of air in the lungs was similar between control and EL lambs. Similarly, while no difference was observed in the LUS images when the lambs were extubated or when the air/oxygen flow rate was decreased, this is not surprising, given only very small decreases in upper tracheal pressure (−1.31 cmH_2_O, *p* < 0.01) and intrapleural pressure (−0.88 cmH_2_O, *p* < 0.01) were detected. A change in the LUS image has been observed in term infants with respiratory distress, utilising a method of providing respiratory support (dual positive airway pressure) which would be expected to result in larger changes in pressure (and therefore more aeration) within the distal airways (22). It is interesting to note that increasing the flow rate had a substantial effect on breathing, often promoting the lambs to sneeze or take irregular breaths (Figure 6) or even inducing apnoeas. While most lambs quickly adjusted to the effects of a high flow rate (such as Figure 6B), and the respiratory rate, effort and variability in breathing were not significantly different 25–30 breaths after the flow rate was changed (Figure 4), in some lambs (such as Figure 6A), irregular breathing patterns or even apnoeas persisted for the entire period that the flow rate was kept at 15 L/min. Taken together, these results suggest that a flow rate of 15 L/min may provide only a minimal increase in end-expiratory pressure and lung aeration, while inducing significant discomfort and possibly reducing spontaneous breathing efforts, particularly in the period immediately after the flow rate is increased.

Backsliding (a deterioration in the appearance of the LUS image) was very common in near-term neonatal lambs, occurring multiple times over the course of the 4 hour experiments in both control and EL lambs. This is consistent with the results of previous observational research (13, 17), where backsliding was observed in over half of both term and preterm infants imaged soon after birth, despite being imaged only 3–4 times. By imaging more often in this experiment, we were able to observe backsliding in nearly every lamb and often observed it multiple times. This significant variation in each LUS image from timepoint to timepoint may reflect a change in lung aeration status (reduction in FRC due to alveolar collapse or liquid reflooding), which may occur when the breathing pattern changes. Indeed, we did notice a reduction in the intrapleural pressure when backsliding was observed in EL lambs, which may reduce the pressure gradient in the airways, leading to airway liquid reflooding. While backsliding may reflect non-uniform aeration and imaging a slightly different region of the lung from scan to scan, care was taken to image the same area between scans. Nevertheless, these are very interesting observations and indicate that lung aeration is not a binary or “all-or-nothing” event whereby the lung transitions from a liquid-filled to air-filled state along a unidirectional path. Instead, it is likely to be a transitionary phase whereby the level of lung aeration increases and decreases at any moment in time, but gradually increases over time as liquid is cleared from lung tissue. The trajectory of this process would appear to be very different in control and EL lambs.

“This study has several limitations. The oxygen/air gas flow rate was only increased to 15 L/min four hours after birth, by which time the lungs were already well aerated, so no change was seen in the LUS images despite a modest increase in the upper tracheal pressure recording. As the lambs were conscious and at times quite active, it was necessary to place them upright in a sling, thereby restricting LUS imaging to a single location on each side of the chest. Furthermore, almost 1,000 ultrasound scans were analysed for this experiment, so it was not feasible to have a second investigator grade all of the images. Nonetheless, the inter-rater reliability for a random sample of 10% of the images was 82%. Additionally, estimating the proportion of air in the LUS images from the CoV of pixel intensity is a largely objective measurement, which is not subject to differences in interpretation between observers.

## 5. Conclusion

LUS was able to monitor lung aeration and liquid clearance over the first four hours after birth in spontaneously breathing near-term lambs and correlated well with changes observed in AaDO_2_. While lung liquid clearance was evident in both groups, the trajectory of liquid clearance was much slower in lambs with elevated liquid levels. However, following lung aeration, backsliding was common in both control and elevated liquid lambs. While increasing the oxygen/air gas flow rate to 15 L/min did not improve lung aeration, these assessments were made at 4 h after birth, when most lambs appeared to have well aerated lungs. New LUS image analysis techniques, such as estimating the proportion of air from the CoV of pixel intensity, may be able to detect small to moderate differences in lung aeration that cannot be readily identified by qualitative scoring systems.

## Data Availability

The raw data supporting the conclusions of this article will be made available by the authors, without undue reservation.
